# On the emergent capabilities of ChatGPT 4 to estimate personality traits

**DOI:** 10.3389/frai.2025.1484260

**Published:** 2025-02-13

**Authors:** Marco Piastra, Patrizia Catellani

**Affiliations:** ^1^Department of Industrial, Computer and Biomedical Engineering, University of Pavia, Pavia, Italy; ^2^Department of Psychology, Catholic University of the Sacred Heart, Milan, Italy

**Keywords:** Large Language Models, ChatGPT, personality traits, Big Five, conversational agents, text analysis

## Abstract

This study investigates the potential of ChatGPT 4 in the assessment of personality traits based on written texts. Using two publicly available datasets containing both written texts and self-assessments of the authors’ psychological traits based on the Big Five model, we aimed to evaluate the predictive performance of ChatGPT 4. For each sample text, we asked for numerical predictions on an eleven-point scale and compared them with the self-assessments. We also asked for ChatGPT 4 confidence scores on an eleven-point scale for each prediction. To keep the study within a manageable scope, a zero-prompt modality was chosen, although more sophisticated prompting strategies could potentially improve performance. The results show that ChatGPT 4 has moderate but significant abilities to automatically infer personality traits from written text. However, it also shows limitations in recognizing whether the input text is appropriate or representative enough to make accurate inferences, which could hinder practical applications. Furthermore, the results suggest that improved benchmarking methods could increase the efficiency and reliability of the evaluation process. These results pave the way for a more comprehensive evaluation of the capabilities of Large Language Models in assessing personality traits from written texts.

## 1 Introduction

Generative Large Language Models (LLM) such as ChatGPT have demonstrated their exceptional ability to generate effective and valuable content in response to a given prompt. Their use as dialogue agents to assist in a variety of everyday tasks has steadily increased. Numerous studies have also highlighted the practical effectiveness of conversational agents powered by LLMs. For example, Matz et al. (2024) showed that ChatGPT can generate persuasive messages tailored to individual psychological characteristics. Furthermore, experimental research presented in Salvi et al. (2024) shows that when given access to personal information, ChatGPT can effectively participate in discussions, leading to high levels of consensus between people on contentious issues.

In this context, the potential to use LLMs in practical applications is extremely appealing. Such applications can range from personalized psychological support and adaptive learning systems to more sophisticated human-computer interactions tailored to the needs, goals, and resources of the interlocutors. In this area, a first major challenge is to use LLMs such as ChatGPT 4 to develop agents that can assess the psychological characteristics of users and adapt their responses accordingly, both in terms of content and style. A second major challenge is to achieve this adaptability without having to retrain or reassess the LLMs when the context or application changes. To address both challenges, in this study we investigated how accurately models such as ChatGPT 4 can assess the psychological characteristics of individuals from written text.

Our study focuses on the Big Five personality dimensions (Costa and McCrae, 1992) because they are widely used in psychology and there is great interest in deriving them automatically from texts. Numerous studies have investigated text analysis using computational linguistics (CL), machine learning (ML), and deep learning (DL) methods (Feizi-Derakhshi et al., 2022; Singh and Singh, 2024). Given the challenges described above, we wanted to investigate the emergent capabilities of ChatGPT 4 in the context of text analysis, i.e., the capabilities that the system exhibits without explicit training for this specific purpose. While ChatGPT 4 has been trained extensively on a huge corpus and has an extremely wide range of information, to our knowledge it has not been trained specifically for the task under consideration here.

To explore these capabilities, we used a “zero-shot” prompting strategy, where the prompt did not contain example scores for traits. We also chose not to use enhancement techniques such as Retrieval-Augmented Generation (RAG) (Lewis et al., 2020). The use of RAG would introduce numerous relevant and variable factors (Gao et al., 2024) and thus significantly expand the scope of the study for systematic evaluation. For similar reasons, we decided not to include multi-step prompting techniques such as Chain-of-Thought (CoT) (Wei et al., 2022).

Many studies, especially in the field of machine learning, have used either binary (‘yes’/‘no’) or three-level scales (‘high’/‘medium’/‘low’) for trait evaluation. This approach was also used with ChatGPT 4 in Ji et al. (2023) and Derner et al. (2023). Binary trait scores are of limited value for practical applications in psychology and may not fully capture the nuances required for practical purposes. In contrast, traditional methods, such as self-assessments or other human evaluations with questionnaires, rely on continuous numerical scales. Consequently, many existing meta-analytical studies on this topic (Moreno et al., 2021; Sun et al., 2024) use the Pearson correlation to compare the results of different methods with those of humans. With this in mind, our study addressed a first research question:

**RQ1**: How do the zero-shot assessments of the Big Five personality traits by ChatGPT 4 compare to other methods when the assessments are conducted with an extended numeric scale?

Another important aspect to consider is the reliability of ChatGPT 4’s estimates. ChatGPT 4 generates quantitative estimates of personality traits when prompted, regardless of the representativeness or length of the analyzed text. Assigning reliable confidence values to each estimate could increase the robustness of practical applications and help determine when additional text is needed to improve the accuracy of the estimates. This led us to a second research question:

**RQ2**: How effectively can ChatGPT 4 assess its own confidence in the estimates it makes, especially in terms of the amount and representativeness of the analyzed text?

In our study, we used two different datasets. The *essays* dataset (Pennebaker and King, 1999) was chosen due to its wide use in computational linguistics and machine learning. This large dataset contains essays written by 2,467 psychology students who were asked to answer a self-assessment questionnaire on the Big Five. The dataset was collected over a period of 7 years, with multiple cohorts of participants and with partially different questionnaires. Therefore, the raw numerical results need to be pre-processed and normalized. The more recent *pan15* dataset (Pardo et al., 2015) was specifically selected for answering our RQ2 regarding ChatGPT 4’s confidence. Each of the 294 participants contributed a substantial number of Twitter messages and this allowed us to evaluate the performance trends of ChatGPT 4 with decreasing amounts of text, by analyzing subsets with different lengths of available messages. In this dataset, the numerical self-ratings for the Big Five traits are reported on a normalized scale.

For the prompting strategy, we defined a baseline prompt where ChatGPT 4 was instructed to act as a social psychologist and provide its assessments in a structured JSON output with numerical ratings. This baseline prompt was then extended to include a prompt to assign a confidence value to each estimate within the JSON structure. We also explored several other variants of the prompt. Given the large and diverse texts on which ChatGPT 4 is trained, we anticipated the possibility of the “Library of Babel” syndrome (Borges, 1998), in which the system might recognize numerous features that are not necessarily relevant to the task. To address this issue, we tested the effectiveness of including different definitions and descriptions of the Big Five in the main prompt.

The remainder of this paper is organized as follows: Section 2 describes the methods used in the experiments, including the datasets, prompting strategies, and evaluation metrics. Section 3 presents the experimental results, followed by a discussion and conclusion in Section 4, which also suggests directions for future research.

## 2 Methods

### 2.1 Datasets

The first dataset utilized in this study is referred to as the *essays* dataset (Pennebaker and King, 1999), which includes data from 2,467 participants. Each participant was instructed to write in a ‘stream of consciousness’ style for 20 min. Participants’ Big Five personality traits were assessed through replies to questionnaires.

Although the essays dataset is widely used in literature, especially in the machine learning field, it presents certain challenges for this task. The dataset includes binary scores (‘yes’/‘no’) for each Big Five personality trait, derived from raw test scores through standardization (shifting by the sample mean and dividing by the standard deviation, then thresholding at zero). However, data were collected between 1997 and 2004 (excluding 2001), and the dataset’s standardization was performed annually, using each cohort’s statistics. Overall, this makes such binary scores unsuitable for this study, since ChatGPT 4 or any machine learning system cannot predict those cohort statistics in a zero-shot modality. In addition, the type of raw scores for the Big Five test in the dataset vary depending on the year of data collection: (1) scores from a 60-item test (1997–1999); (2) average per-item scores (2000 and 2002, number of items unknown); (3) scores from a 44-item test (2002–2004). To create a viable reference for the experiments, we converted all raw test scores into per-item averages, normalized them to a range [−0.5, +0.5] and rounded to the nearest tenth. After removing a few incomplete records, a revised dataset of 2,347 participants was made available.

The second dataset used in our study was the so-called *pan15* dataset (Pardo et al., 2015), which contains data from 294 participants. Participants were asked to provide the researchers with a series of their Twitter messages and each participant allowed a certain number of Twitter messages to be recorded. Most entries (81%) contain exactly 100 messages, while the remaining ones have a count that ranges between 26 and 98 messages. Participants’ Big Five traits were assessed using the BFI-10 test. The dataset contains raw Big Five test scores normalized to a range of [−0.5, +0.5] and rounded to the nearest tenth.

### 2.2 Prompting strategy

For developing our prompting strategy, we adopted the format system + user of the OpenAI platform interface. The system part was used to outline the task, while the user part contained the text to be analyzed. In our experiments, we included different possible combinations of task prompts. Therefore, the system part of the prompt had the following general structure:

$\mathrm{task}\_\mathrm{description}+\left[\mathrm{confidence}\right]+\mathrm{format}+\left[\mathrm{additional}\_\mathrm{description}\right]\text{.}$

The *task_description* section of the prompt was as follows:As a social psychologist, your task is to analyze a written text to assess the author’s big FIVE personality traits(as described in 'A FIVE-factor theory of personality' by Paul Costa and R.R. McCrae).
Your goal is to estimate a score between −0.5, if a trait does not apply, and +0.5, if the trait does apply.


The optional *confidence* section of the prompt was as follows:For each trait, also provide a score in the range of 0.0 to 1.0 to quantify your confidence in the estimate.


The *format* section of the prompt was as follows:Please simply provide your scores in a JSON format, with fields named ["Extraversion", "Neuroticism", "Agreeableness", "Conscientiousness", "Openness"]


If the *confidence* section was also present in the prompt, the following text was inserted into the *format* section:with a substructure containing the two fields ["score", "confidence"] per each of them


Finally, in the optional *additional_description* section we included some theory-based explanations to help ChatGPT 4 focus on the specific type of scoring it should perform. We tested the effectiveness of the following variants of additional descriptions (the actual texts are included in the Supplementary material):

- BFI-44: we included the 44 textual items of the test, categorized as positive or negative for each trait (Costa Mastrascusa et al., 2023).
- BFI-10: we included the 10 textual items of the test, categorized as positive or negative for each trait (Rammstedt and John, 2007).
- Factor Markers: we included the 100 main adjectives described in Goldberg (1992), categorized as positive or negative for each trait.
- Subjective Coding: we included a set of frequently used descriptive words, as described in Ames and Bianchi (2008).
- Profile Facets: we included the six facets describing each trait, as proposed by Roivainen (2016).

The *baseline* version of the *system* prompt did not contain any *additional_description*.

### 2.3 Experiments

All experiments were conducted via an interface with the OpenAI platform using the OpenAI Python package version 1.30.1. Responses were generated from the gpt-4o-2024-05-13 model. The queries were run in batch mode. For all queries, the parameters were set with a temperature value of 0.0 and a seed value of 123. Nevertheless, we noticed some residual fluctuations in the numerical scores across multiple trials. Hence, we conducted each experiment four times and subsequently considered the average values.

The complete Python source code used for the experiments is available in the Supplementary material. It also includes instructions on how to obtain the datasets from their respective owners.

### 2.4 Responses evaluation

Upon request, ChatGPT 4 produced two distinct types of JSON structures, which varied based on whether the *confidence* section was present or absent in the system prompt. If no confidence values were requested, ChatGPT 4 produced responses in the following format:{"Extraversion": 0.2,
"Neuroticism": 0.3,
"Agreeableness": 0.1,
"Conscientiousness": -0.1,
"Openness": 0.3}


When confidence values were requested, Chat GPT produced a response in the format described below:{"Extraversion": {"score": 0.2, "confidence": 0.7},
"Neuroticism": {"score": 0.3, "confidence": 0.8},
"Agreeableness": {"score": 0.1, "confidence": 0.6},
"Conscientiousness": {"score": -0.1, "confidence": 0.7},
"Openness": {"score": 0.3, "confidence": 0.7}}


The structured response format described above was adopted to avoid any uncertainty in the understanding of results, while simultaneously providing the necessary flexibility for additional evaluation and comparison.

ChatGPT 4’s predictive performance was evaluated by comparing its predicted Big Five scores with the actual normalized scores in each of the two datasets. The Root Mean Square Error (RMSE) and Pearson correlation coefficient were calculated for each trait under all the various conditions. Due to the use of a unit scale, all RMSE values coincide with their normalized versions, referred to as NRMSE.

## 3 Results

Table 1 addresses our RQ1 regarding the effectiveness of ChatGPT 4 in predicting the Big Five personality traits from the texts in the two datasets. For each dataset, the first row shows the mean self-report score and the standard deviation. The following four blocks of rows correspond to four experiments: (1) *baseline* prompt; (2) *baseline* prompt with Profile Facets descriptions; (3) *baseline* prompt with *confidence* prompt; and (4) *baseline* prompt with Profile Facets descriptions and *confidence* prompt. Each block reports the mean scores and standard deviations assigned by ChatGPT 4, the numerical accuracy relative to the self-report scores (expressed as 1 – NRMSE), and the Pearson correlation between the ChatGPT 4 scores and the self-report scores. Where applicable, the block also indicates the mean and standard deviation of the confidence values that ChatGPT 4 assigns to its trait estimates. All data reported in Table 1 and in this section are mean values calculated over four repetitions to ensure stability (see Section 2.3).

**Table 1:** Effectiveness of ChatGPT 4 in predicting the Big Five personality traits.

	Description		Extraversion	Neuroticism	Agreeableness	Conscientiousness	Openness
essays	Self-Report	*scores, mean (SD)*	0.107 (0.20)	−0.011 (0.20)	0.175 (0.16)	0.118 (0.15)	0.158 (0.17)
	ChatGPT, baseline	*scores, mean (SD)*	0.079 (0.24)	0.375 (0.11)	0.133 (0.18)	0.087 (0.21)	0.251 (0.14)
		*1 - NRMSE*	0.735	0.567	0.787	0.770	0.790
		*correlation*	**0.276**	0.283	0.239	0.261	0.269
	ChatGPT, facets	*scores, mean (SD)*	0.082 (0.23)	0.342 (0.12)	0.131 (0.18)	0.073 (0.21)	0.233 (0.14)
		*1 - NRMSE*	**0.740**	0.596	0.789	0.774	**0.796**
		*correlation*	0.261	0.282	**0.248**	0.269	**0.271**
	ChatGPT, baseline, confidence	*scores, mean (SD)*	0.076 (0.24)	0.359 (0.11)	0.146 (0.17)	0.128 (0.20)	0.266 (0.12)
		*1 - NRMSE*	0.735	0.582	**0.794**	**0.784**	0.788
		*correlation*	0.270	**0.287**	0.244	**0.281**	0.252
		*confidence, mean (SD)*	0.728 (0.07)	0.819 (0.09)	0.660 (0.09)	0.680 (0.11)	0.705 (0.10)
	ChatGPT, facets, confidence	*scores, mean (SD)*	0.071 (0.23)	0.327 (0.11)	0.140 (0.17)	0.099 (0.20)	0.248 (0.12)
		*1 - NRMSE*	0.734	**0.609**	0.793	0.780	**0.796**
		*correlation*	0.246	0.276	0.238	0.274	0.256
		*confidence, mean (SD)*	0.724 (0.07)	0.809 (0.09)	0.660 (0.10)	0.670 (0.11)	0.697 (0.11)
pan15	Self-Report	*scores, mean (SD)*	0.170 (0.16)	−0.139 (0.23)	0.133 (0.16)	0.173 (0.15)	0.253 (0.15)
	ChatGPT, baseline	*scores, mean (SD)*	0.321 (0.16)	0.077 (0.31)	0.131 (0.19)	0.155 (0.22)	0.375 (0.14)
		*1 - NRMSE*	0.739	0.606	0.763	0.756	0.759
		*correlation*	**0.130**	0.259	0.087	0.196	−0.042
	ChatGPT, facets	*scores, mean (SD)*	0.298 (0.14)	0.061 (0.27)	0.154 (0.18)	0.131 (0.22)	0.371 (0.13)
		*1 - NRMSE*	**0.758**	0.638	0.780	0.768	0.767
		*correlation*	0.117	0.277	**0.159**	**0.264**	**−0.025**
	ChatGPT, baseline, confidence	*scores, mean (SD)*	0.312 (0.14)	0.110 (0.24)	0.147 (0.17)	0.164 (0.19)	0.362 (0.12)
		*1 - NRMSE*	0.752	0.623	0.780	0.784	0.778
		*correlation*	0.104	0.273	0.110	0.219	−0.030
		*confidence, mean (SD)*	0.797 (0.08)	0.697 (0.10)	0.660 (0.10)	0.663 (0.13)	0.793 (0.11)
	ChatGPT, facets, confidence	*scores, mean (SD)*	0.284 (0.15)	0.061 (0.24)	0.149 (0.17)	0.143 (0.20)	0.355 (0.12)
		*1 - NRMSE*	**0.758**	**0.655**	**0.784**	**0.779**	**0.780**
		*correlation*	0.062	**0.279**	0.143	0.245	−0.048
		*confidence, mean (SD)*	0.775 (0.07)	0.690 (0.09)	0.662 (0.10)	0.649 (0.14)	0.791 (0.10)

The first row for each dataset provides statistics on self-reported scores. The following four blocks of rows for each dataset correspond to the different prompting strategies used. For each of the two datasets, best values are in bold.

As shown in Table 1, the agreement between self-report scores and ChatGPT 4 estimated scores varied across traits and datasets. ChatGPT 4 tended to overestimate Neuroticism in the essays dataset, Extraversion in the pan15 dataset, and Openness in both datasets. In the essays dataset, the self-reported scores were moderately correlated with the scores estimated by ChatGPT 4 and showed a similar level of correlation. In contrast, in the pan15 dataset, the correlation was moderate for Neuroticism and Conscientiousness, but lower for other traits. It is important to note that these differences are not obvious if you only use the numerical accuracy metric 1 – NRMSE, which remains relatively high even when the correlation is very low or negative (as seen for Openness in the pan15 dataset).

In contrast, the deviations due to the different prompting strategies were less pronounced. When the confidence values were not prompted, the additional description of the Profile Facets in the prompt tended to produce slightly better results for both datasets. Notably, all estimates changed slightly when confidence values were queried from ChatGPT 4, with slight improvements in some cases. For thoroughness, further experiments were conducted by including other additional descriptions in the prompt, as described in Section 2.2. However, the results were almost identical to those obtained with the baseline prompt alone and are therefore not included in the table.

**Figure 1:**
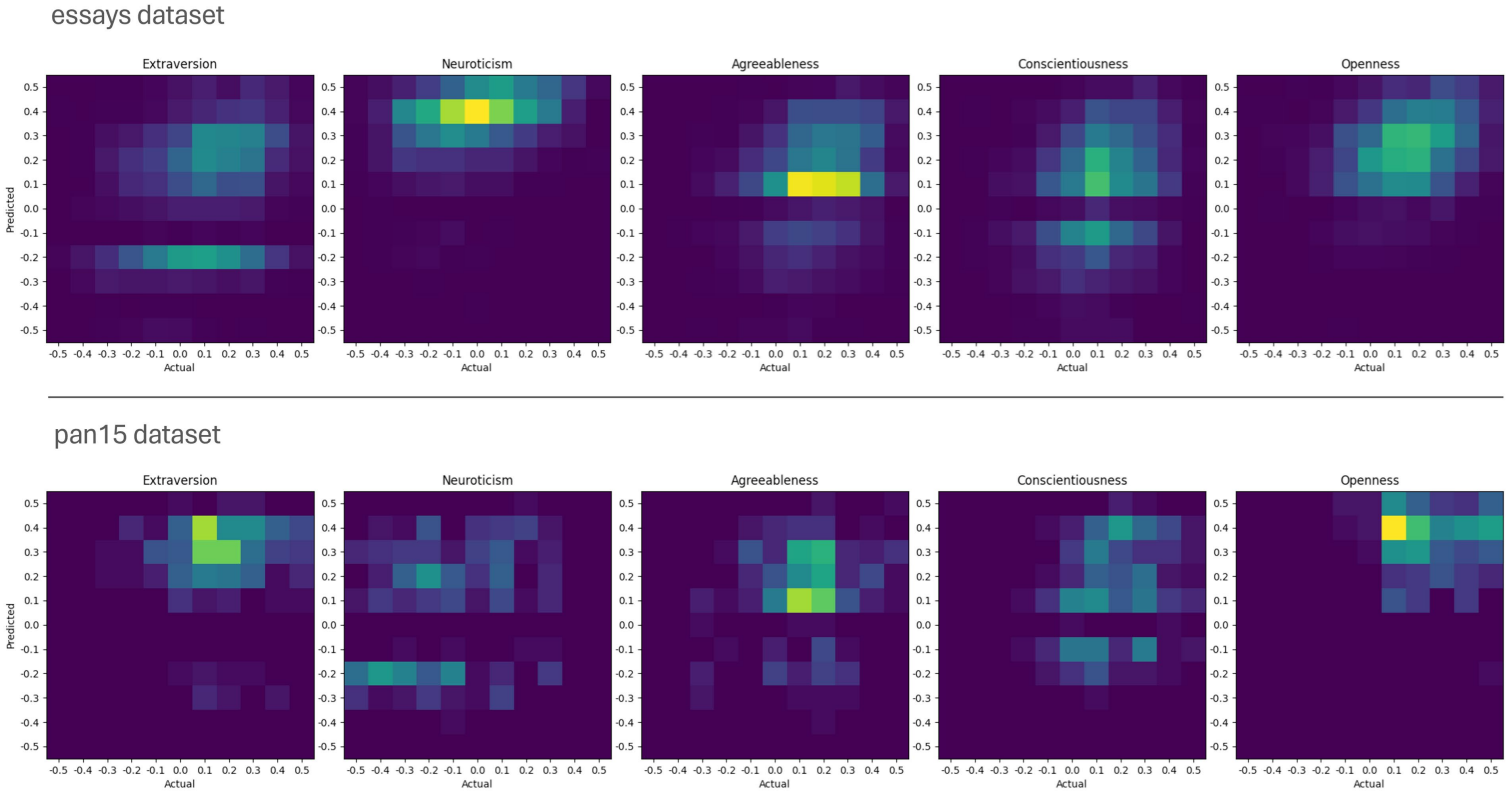
Comparison of ChatGPT 4’s estimated and self-reported scores using 2D histograms. Each histogram shows the frequency of each score combination for each dataset and trait. Self-reported scores are plotted on the x-axis, while ChatGPT 4’s estimates are plotted on the y-axis.

Figure 1 provides additional graphical insights into the comparison between self-report scores and ChatGPT 4 scores using 2D histograms. These histograms illustrate the relative distributions and dispersion of the self-report and estimated scores. In the histograms, the self-report scores are plotted on the x-axis, while the ChatGPT 4 estimates are plotted on the y-axis. The color intensity in each box indicates the number of participants with the corresponding self-reported and estimated scores. The color scales are adjusted independently for each dataset.

The 2D histograms clearly show that in both datasets the self-report scores cluster around certain modes. These modes vary between the different traits but are relatively similar in both datasets. For example, the actual Neuroticism scores in the essays dataset are almost normally distributed around zero, whereas in the pan15 dataset they tend to be scattered around lower scores. As for the scores estimated by ChatGPT 4, the figure shows the frequent presence of a darker band around zero, indicating a systematic bias to avoid zero value estimates. This bias can be seen in every panel except those where all predicted values are above zero (e.g., Openness). Note that, in the pan15 dataset, self-reported scores for Extraversion and Openness, and to a lesser extent for Conscientiousness, are predominantly positive and high. A similar pattern can be observed in the estimated scores of ChatGPT 4. However, as shown in Table 1, the correlation between these scores is low or even negative. Nevertheless, the 1 – NRMSE values remain high due to the numerical proximity of the scores.

**Figure 2:**
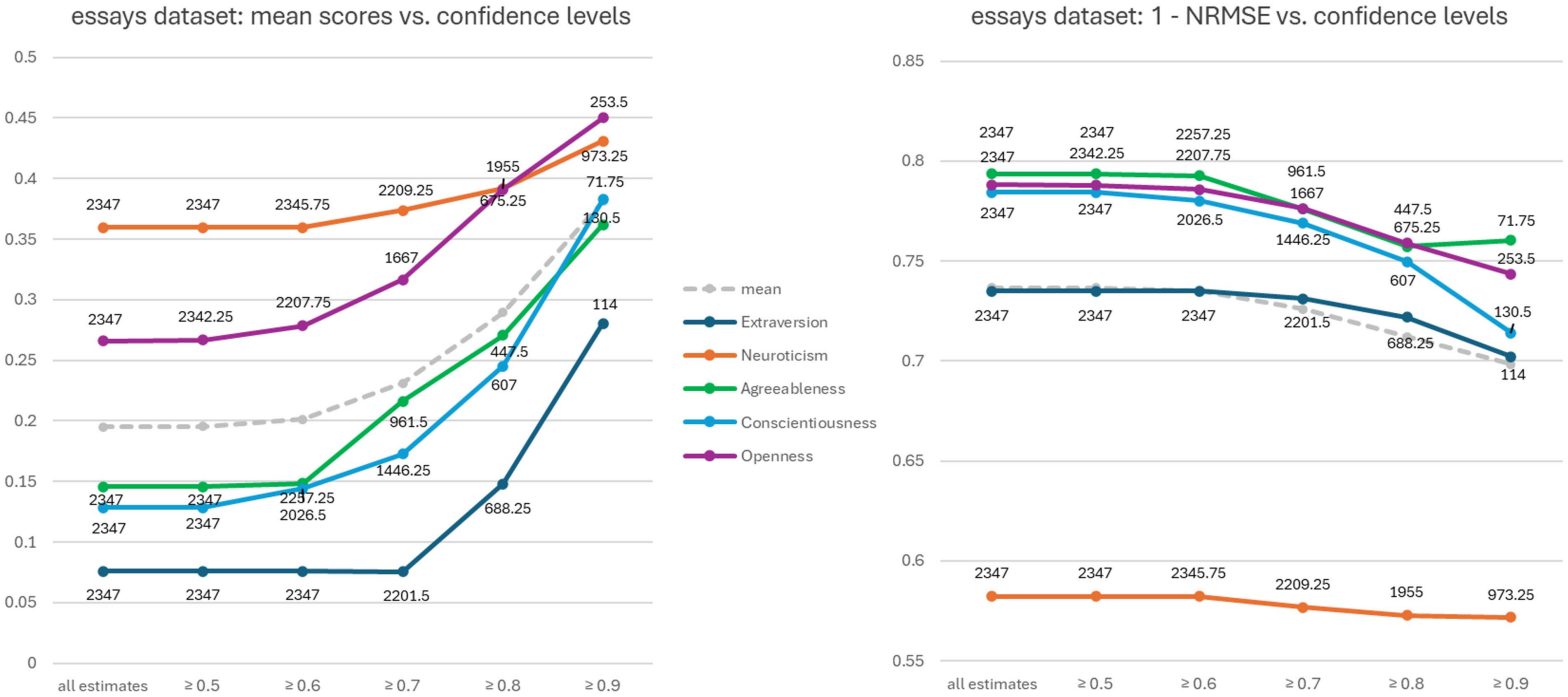
Evaluation of ChatGPT 4’s estimated scores versus its confidence values on the essays dataset. The two diagrams compare confidence values with average estimated scores and 1-NRMSE, respectively.

With regard to our RQ2 on how effectively ChatGPT 4 can assess its confidence in the estimates it makes, Table 1 clearly shows that the confidence values do not correlate with the actual reliability of the estimated trait scores. In the pan15 dataset, for example, the confidence values for Extraversion and Openness are high, while the correlations are not. Figure 2 shows two graphs relating to the essays dataset. In both cases, only the scores estimated by ChatGPT 4 with corresponding confidence values above a certain threshold were considered. The average number of estimated scores with confidence values equal or above the threshold (x-axis) is shown next to each marker.

The graphs shown in Figure 2 illustrate the extent to which ChatGPT 4 confidence values are related to its estimates. The graph on the left-hand side of Figure 2 shows that ChatGPT 4 confidence values correlate well with the mean of the estimates, suggesting that ChatGPT 4 tends to have more confidence in higher estimates. However, the graph on the right-hand side of Figure 2 shows that this increased confidence is not accompanied by an improvement in numerical accuracy, but rather the opposite.

**Figure 3:**
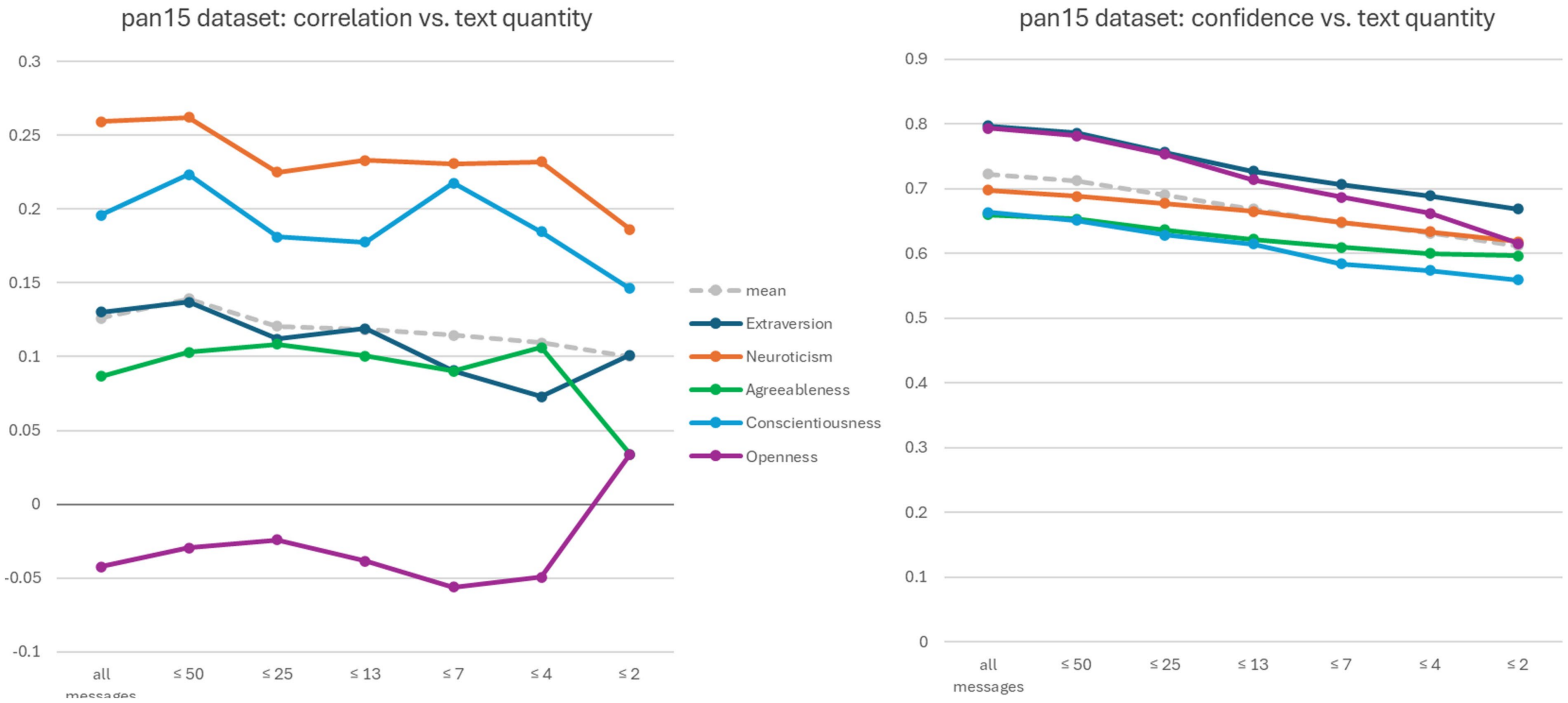
Evaluation of ChatGPT 4’s confidence values versus text amount on the pan15 dataset. The two diagrams compare text amounts with the correlation between estimated and self-reported scores, and with confidence values, respectively. The x-axis represents the thresholds applied in terms of the number of Twitter messages.

Figure 3 shows two graphs related to the pan15 dataset. Several experiments were conducted in which the amount of text provided to ChatGPT 4 for analysis was gradually reduced. Specifically, while maintaining the same system prompt (see Section 2.2), the user prompt was created by selecting the first *n* Twitter messages recorded for each participant. The decreasing *n* values are shown on the x-axis in both graphs.

The graph on the left-hand side of Figure 3 shows that the correlation between ChatGPT 4 estimated and self-reported scores tends toward zero for all traits as the amount of text decreases, which is to be expected. The graph on the right-hand side of Figure 3 shows that although the confidence values decrease, they remain relatively high for all traits, even when the correlation tends toward zero (as seen for Conscientiousness and Openness).

## 4 Discussion and conclusions

Our study showed the promising abilities of ChatGPT 4 in estimating personality traits from written texts (RQ1), while the confidence values of ChatGPT 4 were not well related to numerical accuracy (RQ2).

Regarding the ability of ChatGPT 4 to assess personality traits, we found that the best correlation values of ChatGPT 4 were between 0.25 and 0.29 (Table 1). In a meta-analysis by Moreno et al. (2021), the authors summarized 23 independent studies on the correlations between the Big Five personality traits and computational indicators derived from written language and found correlation coefficients between 0.26 and 0.30 (which were considered small to moderate effect sizes). Another meta-analysis by Sun et al. (2024) of 26 studies on the same task found correlation coefficients between 0.29 and 0.40. From this, we can conclude that ChatGPT-4’s ability to assess the Big Five personality traits is remarkable, especially considering that no explicit training was performed for this specific task.

A less satisfactory result was obtained about the ability to reliably assign confidence values to the numerical ratings. We found that asking for confidence scores had a slight impact on the predicted scores, and not always for the better. As can be seen in Figure 2, ChatGPT 4’s confidence scores were not well related to numerical accuracy, even for the same traits. And Figure 3 shows that ChatGPT 4 is unaware of the unreliability of its estimated scores, even when the text is clearly insufficient for credible analysis. These combined factors could significantly hinder real-world applications, as it would be difficult to determine whether the input text is adequate or representative enough to draw accurate conclusions based on ChatGPT 4’s scores alone. Therefore, improving this form of capability should be an important goal for future research.

The results of our study suggest that the methodology used to determine whether ChatGPT can perform comparably to other methods in inferring the Big Five needs further refinement. For example, the benchmark datasets should be improved. Binary trait scores are of limited value for practical applications in psychology, and more accurate numerical scores should be used. In this study, we made progress in this direction, and subsequent studies may make further progress in other directions. In particular, the assessment of ChatGPT 4 ability was very often done, as we did, by comparing the scores estimated by ChatGPT 4 with the self-assessments of the people who wrote the texts. It might make more sense to compare the scores estimated by ChatGPT with other scores resulting from similar assessments by human judges. In this way, we would avoid comparing an observer’s scores with those of the person themselves, introducing additional errors that are not necessarily related to the predictor’s abilities.

As a limitation, it must be acknowledged that while focusing on the zero-shot approach allows for a more manageable study, it may not capture the full potential of ChatGPT 4 when supplemented with additional fine-tuning or techniques such as RAG or CoT.

To summarize, the emerging capabilities of ChatGPT 4 in inferring personality traits from texts seem promising. However, to assess its actual reliability, further studies should be conducted using a range of methodological strategies and procedures such as the one presented here. With the ultimate goal of developing a common method by which we can best test the possibilities of using ChatGPT and other LLM models in the psychological assessment of people.

## Data Availability

The data analyzed in this study is subject to the following licenses/restrictions: datasets can be obtained from respective owners, as described in the Supplementary material. Requests to access these datasets should be directed to James W. Pennebaker, https://liberalarts.utexas.edu/psychology/faculty/pennebak; https://pan.webis.de/clef15/pan15-web/author-profiling.html.
